# Mercury Ion Binding to Apolipoprotein E Variants ApoE2, ApoE3, and ApoE4: Similar Binding Affinities but Different Structure Induction Effects

**DOI:** 10.1021/acsomega.2c02254

**Published:** 2022-08-12

**Authors:** Elina Berntsson, Merlin Sardis, Andra Noormägi, Jüri Jarvet, Per M. Roos, Vello Tõugu, Astrid Gräslund, Peep Palumaa, Sebastian K. T. S. Wärmländer

**Affiliations:** † Department of Chemistry and Biotechnology, Tallinn University of Technology, 12618 Tallinn, Estonia; ‡ Department of Biochemistry and Biophysics, Stockholm University, 106 91 Stockholm, Sweden; § The National Institute of Chemical Physics and Biophysics, 12618 Tallinn, Estonia; ∥ CellPept Sweden AB, Kvarngatan 10B, 118 47 Stockholm, Sweden; ⊥ Institute of Environmental Medicine, Karolinska Institutet, 171 77 Stockholm, Sweden; # Department of Clinical Physiology, Capio Saint Göran Hospital, 112 19 Stockholm, Sweden

## Abstract

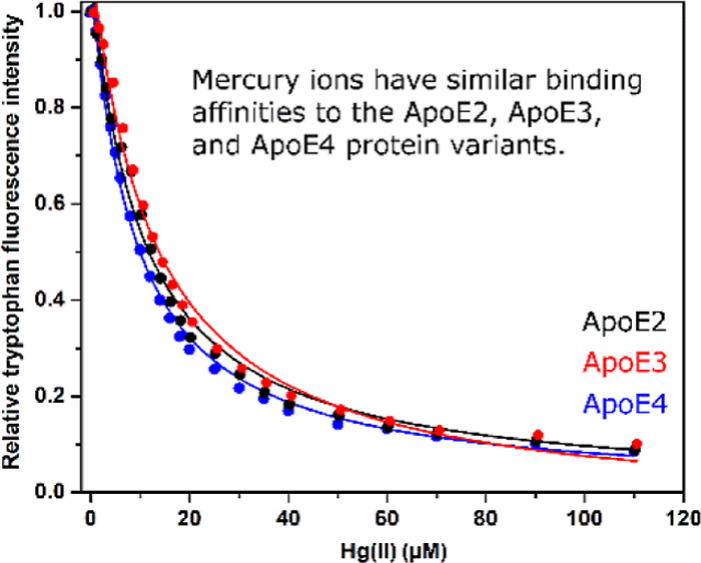

Mercury intoxication typically produces more severe outcomes in people with the *APOE-*ε*4* gene, which codes for the ApoE4 variant of apolipoprotein E, compared to individuals with the *APOE-*ε*2* and *APOE-*ε*3* genes. Why the *APOE-*ε*4* allele is a risk factor in mercury exposure remains unknown. One proposed possibility is that the ApoE protein could be involved in clearing of heavy metals, where the ApoE4 protein might perform this task worse than the ApoE2 and ApoE3 variants. Here, we used fluorescence and circular dichroism spectroscopies to characterize the *in vitro* interactions of the three different ApoE variants with Hg(I) and Hg(II) ions. Hg(I) ions displayed weak binding to all ApoE variants and induced virtually no structural changes. Thus, Hg(I) ions appear to have no biologically relevant interactions with the ApoE protein. Hg(II) ions displayed stronger and very similar binding affinities for all three ApoE isoforms, with *K*_D_ values of 4.6 μM for ApoE2, 4.9 μM for ApoE3, and 4.3 μM for ApoE4. Binding of Hg(II) ions also induced changes in ApoE superhelicity, that is, altered coil–coil interactions, which might modify the protein function. As these structural changes were most pronounced in the ApoE4 protein, they could be related to the *APOE-*ε*4* gene being a risk factor in mercury toxicity.

## 1 Introduction

Mercury (Hg) is a toxic metal that contributes to severe and permanent health problems and even death.^1−4^ According to the Global Mercury Assessment 2018 report,^5^ the estimated global anthropogenic emission of mercury to the atmosphere was approximately 20% higher in 2015 than in 2010. Most of these emissions originate from industrial activities related to mining and coal and oil combustion,^5^ where Asia is responsible for 49% of the total emissions, followed by South America (18%) and Sub-Saharan Africa (16%). Hg is neurotoxic and genotoxic and induces damage to organs such as the brain and kidneys.^1,2,6^ The different forms of Hg, that is, metallic, inorganic such as Hg(I), and Hg(II) ions, and organometallic complexes such as methyl-Hg and ethyl-Hg, have different properties and toxicity profiles.^1,2^ Mercury vapor and organic Hg readily pass through membranes such as the blood–brain–barrier and the placental barrier, and thus become distributed throughout the entire human body including the fetus.^7−9^ Developing neurites^10^ and growing organs seem to be particularly susceptible to Hg damage, and Hg exposure is therefore especially harmful for children and fetuses.^11−15^ The molecular mechanisms underlying Hg toxicity remain unclear,^16^ but appear to include toxic molecular mimicry^8^ and blocking of antioxidants^17^ especially in the mitochondria.^18^

Interestingly, the susceptibility to Hg toxicity is influenced by genetic factors.^19,20^ Notably, Hg exposure has been found to produce more severe outcomes in people with the *APOE-*ε*4* gene, which codes for the ApoE4 version of the apolipoprotein E protein, compared to individuals with the *APOE-*ε*2* and *APOE-*ε*3* genes.^6,12−14,19,21−23^*APOE-*ε*4* is also a genetic risk factor for Alzheimer’s disease (AD),^21,24−29^ and likely for other proteinopathies as well,^30,31^ while Hg exposure might be an environmental risk factor for AD.^28,32−35^ Why *APOE-*ε*4* carriers are more susceptible to both mercury intoxication and AD remains unclear. A number of possible explanations have been proposed,^28,36,37^ including the possibility that the ApoE protein might be involved in the clearance of Hg and/or of amyloid-β (Aβ) peptides,^21,27,38^ whose aggregation plays a central role in AD pathology.^39,40^

**Figure 1:**
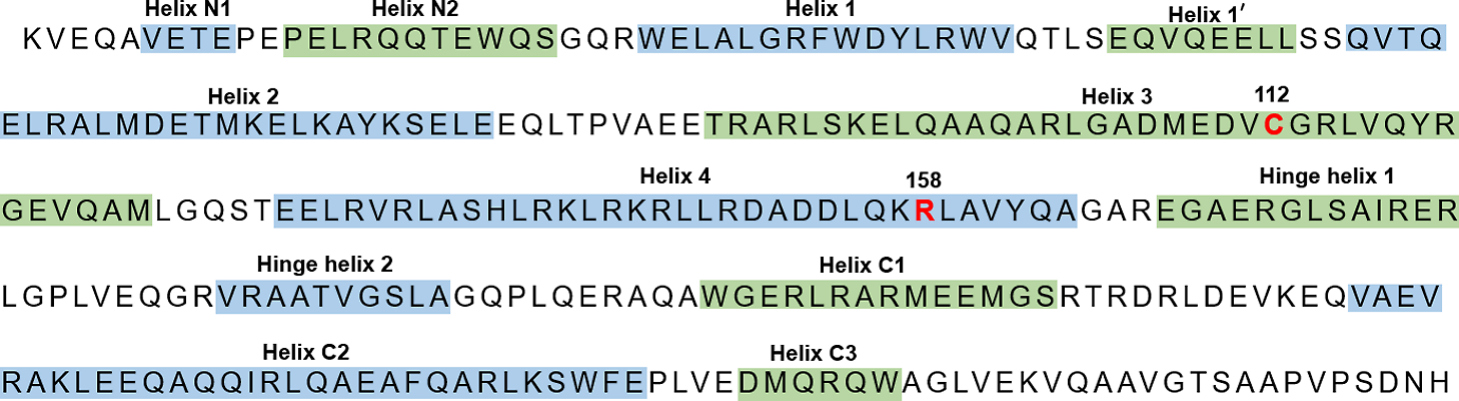
Primary structure of the apolipoprotein E3 protein. The ApoE2 (Cys112 and Cys158), ApoE3 (Cys112 and Arg158), and ApoE4 (Arg112 and Arg158) variants differ in positions 112 and 158, marked in red. The regions known to adopt α-helix conformations are shown in blue and green based on the information in Chen *et al.* 2011.^49^

Apolipoprotein E (ApoE) is a 299-residue-long (34 kDa) glycoprotein (Figure 1) involved in lipid metabolism: it transports lipid-soluble vitamins and lipids such as cholesterol in the central nervous system, into the lymph system, and then into the blood.^41−43^ In the brain, ApoE is mainly produced by the astrocytes and microglial cells and interacts with ApoE receptors.^38,44^ The three common variants of the ApoE protein differ at residues 112 and 158, that is, ApoE2 (Cys112 and Cys158), ApoE3 (Cys112 and Arg158), and ApoE4 (Arg112 and Arg158).^22,42^ As the cysteine −SH groups are capable of binding metal ions including Hg ions,^35,45−47^ it has been speculated that the ApoE residues Cys112 and Cys158 might bind Hg ions, which subsequently could be transported out from the tissue.^6,21,22,28,46,48^ ApoE4 would then not be able to perform this task very well as it has Arg instead of Cys residues at positions 112 and 158 (Figure 1). Mercury would then accumulate in the tissues of *APOE-*ε*4* individuals, which would aggravate the toxic effects, which possibly could include Hg-induced neurodegeneration and AD.^6^ To the best of our knowledge, no one has so far tested this hypothesis experimentally. Here, we use the biophysical techniques circular dichroism (CD) and fluorescence spectroscopy to study *in vitro* the binding interactions between inorganic Hg(I) and Hg(II) ions and the three different ApoE protein variants.

## 2 Materials and Methods

### 2.1 Reagents and Sample Preparation

Lyophilized ApoE2, ApoE3, and ApoE4 protein variants were purchased from AlexoTech AB (Umeå, Sweden) and stored at −20 °C. All proteins were produced recombinantly with an additional methionine residue in position-1. The integrity of the proteins was confirmed by MALDI-TOF mass spectrometry. Before measurements, samples were dissolved in 10 mM NaOH (prepared in Milli-Q water) according to the manufacturer’s instructions and then allowed to equilibrate at 4 °C for at least 1 h. The concentration was initially determined by weight and then confirmed by UV–vis spectroscopy. The samples were then diluted in either sodium phosphate buffer or MES [2-(*N*-morpholino)ethane sulfonic acid] buffer to a final buffer concentration of 20 mM at pH 7.3 or pH 5.5.

### 2.2 Fluorescence Spectroscopy

The binding affinities between Hg ions and ApoE variants were evaluated by fluorescence measurements using a LS-55 fluorescence spectrophotometer (PerkinElmer Inc., Waltham, MA, USA) equipped with a magnetic stirrer. In order to study both Hg(I) and Hg(II) ions, some measurements were carried out in the presence of 1 mM of the reducing agent TCEP [tris(2-carboxyethyl)phosphine], which reduces the Hg(II) ions from the HgCl_2_ salt to the Hg(I) form. Small aliquots of HgCl_2_ (stock concentrations of 1, 2, or 10 mM) were titrated to a sample containing either 0.2 or 1.0 μM ApoE protein in 20 mM MES buffer, pH 7.3 or 5.5, at 25 °C, in quartz cuvettes with a 5 mm path length. After each addition of HgCl_2_, the solution was stirred for 30 s before recording fluorescence emission spectra at 350 nm (excitation 276 nm). All titrations were repeated 3 times. The measured tryptophan fluorescence intensities were plotted against the concentration of Hg ions, and dissociation constants (*K*_D_) were evaluated by fitting the data curves to either eq 1 (the isotherm or hyperbolic equation together with a Hill coefficient)^50^ or eq 2 (the Morrison equation)^51^
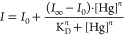
1
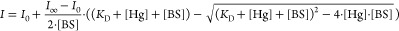
2Here, *I*_0_ is the initial fluorescence intensity with no added Hg ions, *I*_∞_ is the steady-state intensity at the end of the titration, [Hg] is the concentration of the added Hg ions, *K*_D_ is the dissociation constant, *n* (in eq 1) is the Hill coefficient, and [BS] (in eq 2) is the concentration of the binding sites, which is equal to the protein concentration if the protein has a single binding site. Adding a term for the fluorescence quenching effect of free Hg ions appeared unnecessary.^52^

### 2.3 CD Spectroscopy

CD spectra were recorded between 190 and 260 nm with a step size of 0.5 nm and 5 s per data point at 20 °C using a Chirascan CD spectrometer from Applied Photophysics, UK. Samples of 5 μM ApoE protein in 20 mM sodium phosphate buffer, pH 7.3, were measured in a quartz cuvette with an optical path length of 2 mm. Sodium phosphate buffer was used to avoid the interference of MES buffer with the CD measurements. In order to study both Hg(I) and Hg(II) ions, some measurements were carried out in the presence of 1 mM of the reducing agent TCEP, which reduces the Hg(II) ions from the HgCl_2_ salt to the Hg(I) form. After the initial measurement of ApoE alone, 1–2 μL HgCl_2_ was added in steps from stock concentrations of 1, 2, or 10 mM to produce final HgCl_2_ concentrations of 2, 5, 40, and 80 μM. All titrations were repeated two or three times.

## 3 Results and Discussion

### 3.1 Fluorescence Spectroscopy

To determine the binding strength of Hg(I) and Hg(II) ions to the ApoE isoforms, the quenching effect of the ions on the intrinsic tryptophan fluorescence was monitored. ApoE contains seven tryptophan residues (Figure 1), which exhibit a strong fluorescence signal at 350 nm when excited around 276 nm.

**Figure 2:**
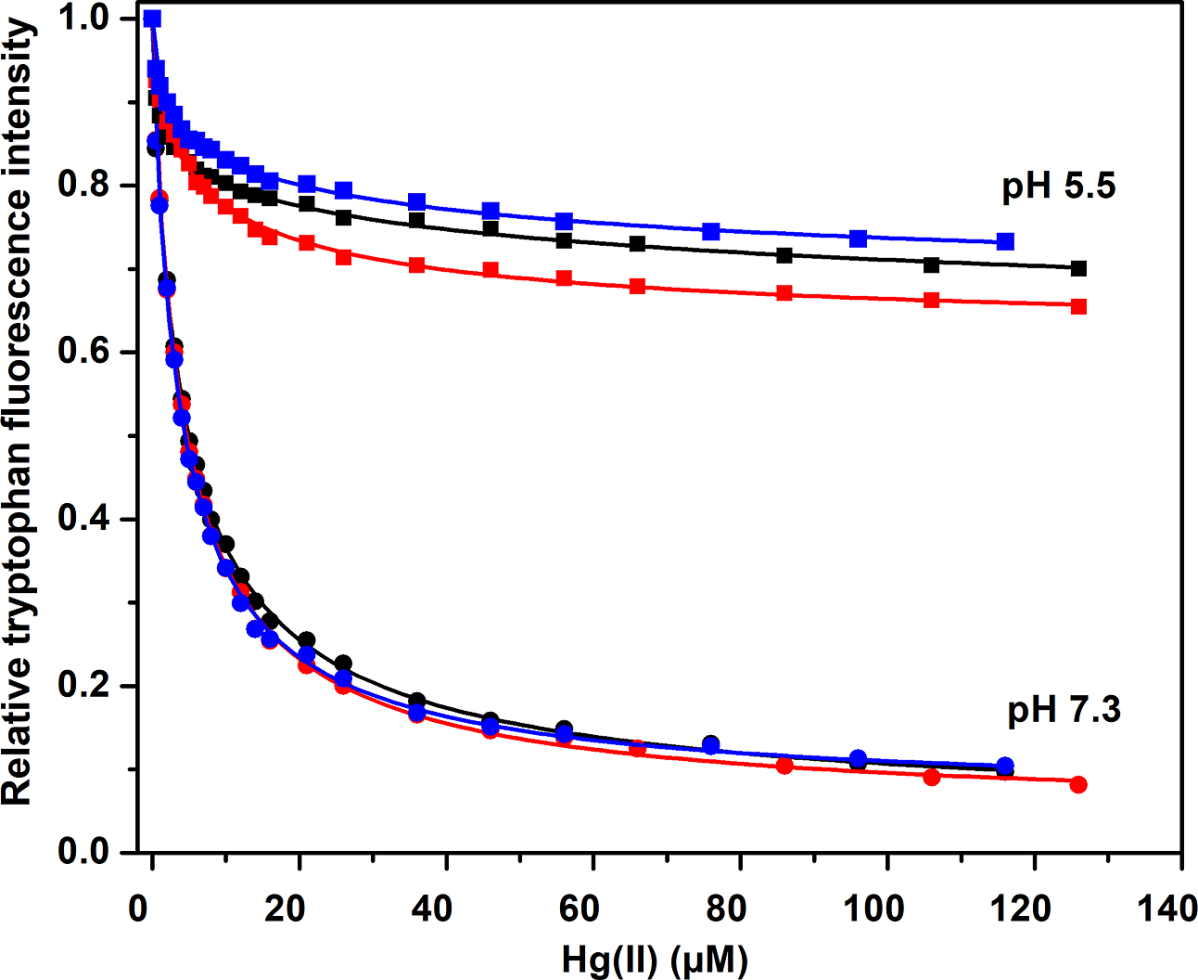
Intrinsic ApoE tryptophan fluorescence upon titration with HgCl_2_ recorded at 350 nm (excitation 276 nm) for 0.2 μM ApoE protein at +25 °C in 20 mM MES buffer either at pH 7.3 or pH 5.5. Black—ApoE2; red—ApoE3; blue—ApoE4; circles—pH 7.3; and squares—pH 5.5. Fitting eq 1 to the titration data produces apparent dissociation constants (*K*_D_^app^).

Titrating HgCl_2_ to 0.2 μM protein samples at either pH 7.3 or pH 5.5 produced the binding curves shown in Figure 2. Fitting eq 1 to the pH 7.3 curves yielded the dissociation constants (*K*_D_) for Hg(II) binding shown in Table 1, that is, on average 4.62 μM for ApoE2, 4.89 μM for ApoE3, and 4.32 μM for ApoE4. The Hill coefficients (*n* in eq 1) were all found to be around 1, indicating little or no binding cooperativity. The hyperbolic equation (*i.e.*, eq 1) was used as the protein concentration clearly was lower than the binding affinity of the studied complex. Even though MES is a good buffer devised to have minimal interactions with metal ions and other cations,^53^ no corrections were made for potential interactions between the buffer and the Hg(II) ions. Thus, the calculated *K*_D_ values in Table 1 should be considered as apparent, that is, *K*_D_^app^. The most important thing about these *K*_D_^app^ values is that they (and the corresponding binding curves) are all very similar. We therefore conclude that the Hg(II) ions are bound by the same (or at least very similar) binding sites in all three ApoE variants and that these binding sites do not involve as binding ligands the residues 112 and 158, which vary between the protein isoforms (Figure 1).

**Table 1:** Apparent *K*_D_ Values (*K*_D_^app^) in μM for the ApoE·Hg(II) Complex Obtained by Fitting Eq 1 to the pH 7.3 Fluorescence Titration Curves Shown in Figure 2

	titration 1	titration 2	titration 3	average *K*_D_
ApoE2	4.54 ± 0.29	4.33 ± 0.36	5.00 ± 0.44	4.62
ApoE3	4.65 ± 0.32	5.11 ± 0.27	4.90 ± 0.22	4.89
ApoE4	4.33 ± 0.35	4.33 ± 0.42	4.29 ± 0.23	4.32

These conclusions clearly contradict the previously suggested hypothesis that different binding affinities to mercury ions could explain why the *APOE-*ε*4* gene is a risk factor in Hg intoxication but not the *APOE-*ε*2* and *APOE-*ε*3* genes.^6,21,22,28,46,48^ However, it cannot be ruled out that other forms of mercury, such as organic methyl-Hg or ethyl-Hg, could display different binding properties to the different ApoE variants. Future studies might investigate the details of ApoE binding to other forms of Hg than the inorganic ions studied here.

The HgCl_2_ titrations at pH 5.5 produced a much lower reduction in Trp fluorescence intensity than the titrations at pH 7.3 (Figure 2). This strongly indicates weaker binding of Hg(II) ions at acidic pH, even though no *K*_D_ values could be derived from these curves. The main difference in the proteins between pH 7.3 and pH 5.5 is protonation of His residues, which have p*K*_a_ values around 6.5.^54^ As His residues are previously known to bind a range of metal ions, including Hg(II) ions,^32^ and as protonation of His residues lowers their affinity for positively charged molecules, the different titration results at pH 7.3 and pH 5.5 shown in Figure 2 strongly suggest that His residues are involved in coordinating the Hg(II) ions. As shown in Figure 1, the ApoE proteins have two His residues, that is, His140 and His299.

**Figure 3:**
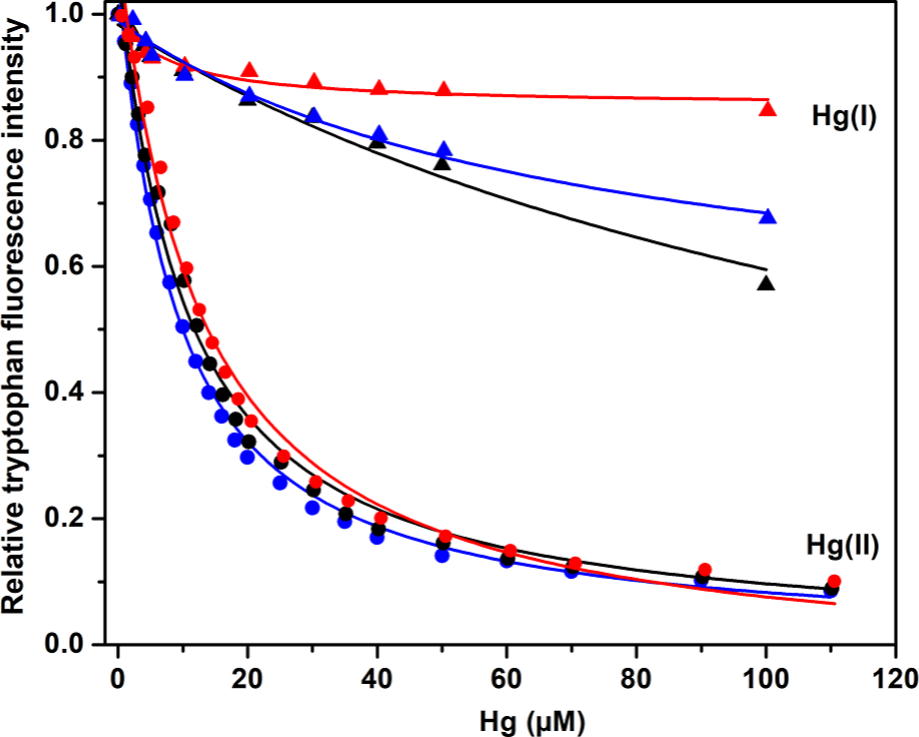
Intrinsic ApoE tryptophan fluorescence upon titration with HgCl_2_ recorded at 350 nm (excitation 276 nm) for 1 μM ApoE protein in 20 mM MES buffer, pH 7.3 at +25 °C. Black—ApoE2; red—ApoE3; blue—ApoE4; circles—Hg(II) ions; and triangles—Hg(I) ions (1 mM TCEP added). Fitting eq 2 to the titration data produces apparent dissociation constants (*K*_D_^app^).

Titrations with HgCl_2_ at pH 7.3 were also carried out at a slightly higher protein concentration, that is, 1.0 μM (Figure 3). As the protein concentration is now close to the binding affinity, it is not appropriate to fit the binding curves with the hyperbolic/isotherm eq 1. The data was instead fitted with eq 2, where the binding site concentration is included as one of the fitted parameters. For the Hg(II) ion titrations, eq 2 produced average *K*_D_ values around 4.2 (ApoE2), 4.1 (ApoE3), and 4.8 (ApoE4). These *K*_D_ values are very close to those obtained at 0.2 μM ApoE concentration (Figure 2 and Table 1), thus confirming their accuracy. The fittings to eq 2 also produced binding site concentrations around 10 μM, for all three proteins. Although this should be regarded as an approximate number, it is clearly larger than the protein concentration of 1 μM. This indicates that there are multiple binding sites for Hg(II) ions on all three ApoE variants.

Adding 1 mM of the reducing agent TCEP reduces the Hg ions to their monovalent Hg(I) state. As shown in Figure 3, titrations with Hg(I) ions yield much lower Trp fluorescence reduction than titrations with Hg(II) ions for all three ApoE variants. It was not possible to fit a binding equation to the Hg(I) data, but the binding of Hg(I) ions appears to be weak—likely in the millimolar range. It is however possible that Hg(I) ions may not quench Trp fluorescence as efficiently as Hg(II) ions, and the Hg(I) titration results should therefore be interpreted with caution.

### 3.2 CD Spectroscopy

**Figure 4:**
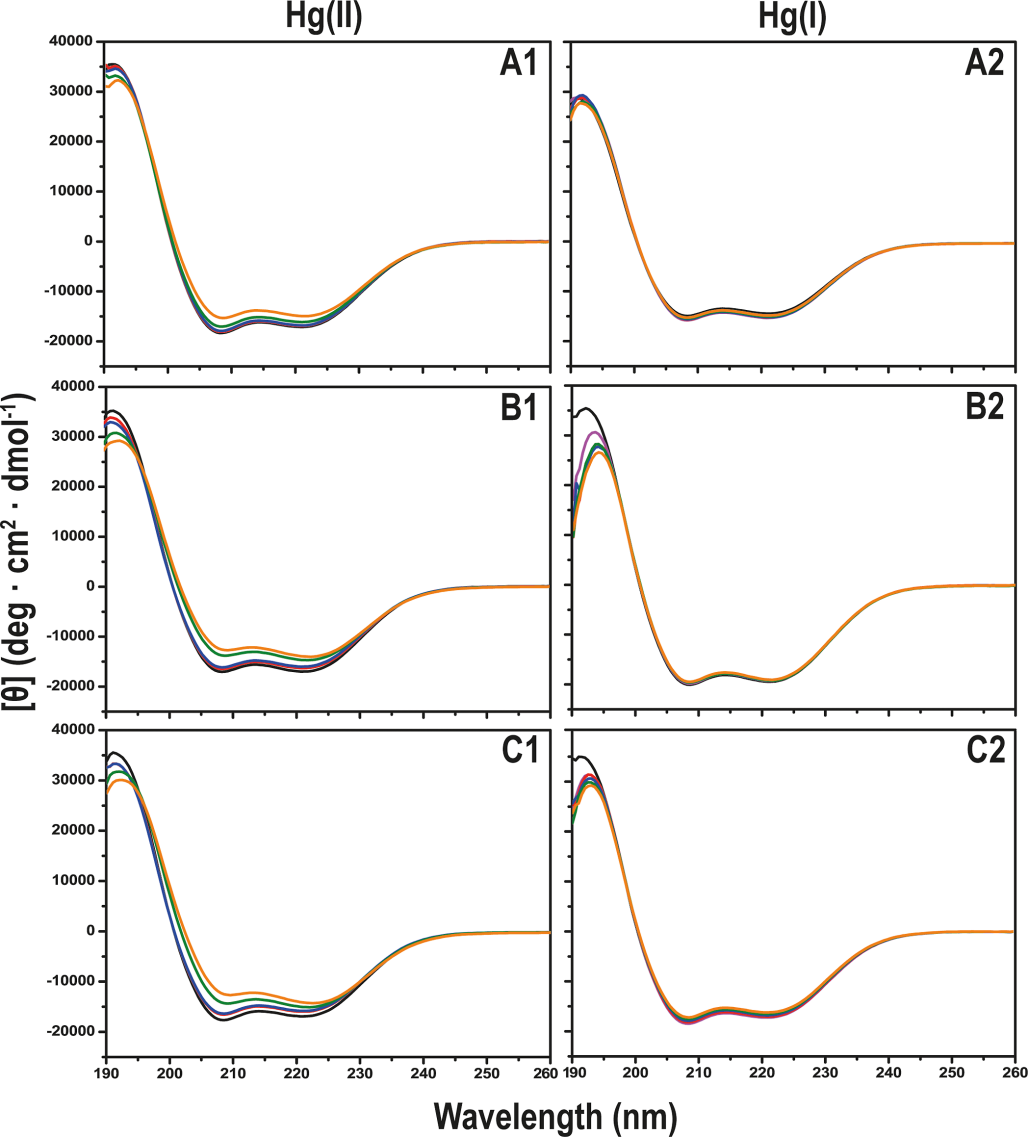
CD spectra showing titrations of 5 μM ApoE protein with HgCl_2_ in 20 mM sodium phosphate buffer, pH 7.3 at 20 °C. Row A: ApoE2; row B: ApoE3; and row C: ApoE4; column 1: Hg(II) ions and column 2: Hg(I) ions (1 mM TCEP added, pink). HgCl_2_ was added in steps of 0 μM (black), 2 μM (red), 5 μM (blue), 40 μM (green), and 80 μM (orange).

ApoE is previously known to be a mainly α-helical protein in aqueous solutions.^49,55^ This was confirmed by our CD measurements, where all three ApoE variants exhibit CD spectra with characteristic minima around 208 and 222 nm (Figure 4), which are typical for α-helical secondary structures.^55^ Upon titration with Hg(II) ions, the CD spectra gradually lost some intensity, although they generally maintained α-helical shapes. These changes cannot be explained by dilution of the sample, as the total increase in volume during the titrations did not exceed 2%.

When titrated with Hg(II) ions, the three ApoE variants showed different intensity losses for the 208 and 222 nm minima (Figure 4 and Table 2). The [θ_222_]/[θ_208_] ratio in CD spectra has previously been shown to reflect hydrophobic coil–coil interactions of α-helical secondary structures, also known as α-helical supercoiling or simply superhelicity.^56−58^ The largest increase in the [θ_222_]/[θ_208_] ratio, indicative of increased helix supercoiling upon addition of Hg(II) ions, is observed for the ApoE4 variant, which changes from 0.96 to 1.16 (Table 2). ApoE2 displays the smallest change, from 0.93 to 0.98, while ApoE3 increases from 0.99 to 1.13 (Table 2). A careful investigation of the ApoE4 CD spectra furthermore shows that the minima display small but clear changes during the titration: in the presence of 80 μM Hg(II) ions, the minima have moved from, respectively, 208.5 to 209.5 nm and from 221 to 222.5 nm (Figure 4(C1)), which is a further indication of secondary structure alterations. All three repetitions of this titration gave the same results. For the ApoE2 variant, no such shifts were observed (Figure 4(A1)). These changes are in line with our previous observations that metal ions can promote protein supercoiling,^59^ and also with earlier reports that Hg(II) ions can generally affect protein folding, misfolding, and aggregation.^60−62^ A previous study showed that the ApoE C-terminal domain, which contains lipoprotein binding and ApoE self-association sites, has an intrinsic propensity to form coil-coiled interactions stabilized by salt bridges.^63^ Thus, it is possible that the observed Hg(II)-induced structural changes take place in the C-terminal domain. Future research should be able to identify the exact location(s) of the Hg(II) binding site(s), the nature of the structural changes, and if binding of Hg(II) ions affects the protein’s function(s).

**Table 2:** Negative CD Signal Intensities [θ × 10^–3^] at 208 and 222 nm together with the Calculated [θ_222_]/[θ_208_] Ratios Derived from the CD Spectra Shown in Figure 3

	ApoE2					ApoE3					ApoE4				
Hg(II) (μM)	0	2	5	40	80	0	2	5	40	80	0	2	5	40	80
θ at 208 nm	18.4	18.1	17.9	17.0	15.2	17.3	16.6	16.2	13.7	12.6	17.6	16.5	16.3	14.2	12.3
θ at 222 nm	17.0	16.8	16.8	16.0	14.9	17.1	16.4	16.2	14.9	14.2	16.8	15.9	15.7	15.0	14.2
[θ_222_]/[θ_208_]	0.93	0.93	0.94	0.94	0.98	0.99	0.99	1.0	1.08	1.13	0.96	0.96	0.96	1.06	1.16

Titrations with monovalent Hg(I) ions, that is, with 1 mM TCEP reducing agent added to the samples, produced much smaller changes in the CD spectra (Figure 4), indicating weaker ApoE binding affinity for Hg(I) ions than for Hg(II) ions. These results are in line with those from the fluorescence measurements (Figure 4), where Hg(I) ions for all three ApoE variants produced much weaker Trp fluorescence reduction than Hg(II) ions. However, it cannot be ruled out that the monovalent Hg(I) ions might have different binding sites and/or different binding configurations than Hg(II) ions and may therefore not induce the same structural changes as Hg(II) ions. Overall though, it appears that the Hg(I) interactions with ApoE proteins are weak and of little biological relevance. As 1 mM TCEP roughly corresponds to the reducing environment inside human cells, our results suggest that biologically relevant interactions between inorganic Hg ions and ApoE proteins will not take place intracellularly, but rather in the oxidizing extracellular environment, where the Hg ions are in their divalent Hg(II) state.

It should be noted that already before addition of the HgCl_2_ salt, there are slight differences in the CD spectra and in superhelicity between the three ApoE variants (Figure 4 and Table 2), indicating different secondary structures. It has previously been suggested that the ApoE variants could exhibit different secondary structures due to the mutations in positions 112 and 158. Specifically, it has been proposed that the N- and C-termini would be positioned closer to each other in ApoE4, than in ApoE2 and ApoE3, due to the interaction between Arg61 and Glu255 in ApoE4.^64,65^ Such an interaction is considered unlikely in ApoE2 and ApoE3, where the Arg61 residue is believed to interact with Cys112 and therefore be unavailable for interactions with Glu255. Given the different initial structures, it is not surprising that addition of Hg(II) ions appears to induce somewhat different structural alterations in the three variants.

## 4 Conclusions

Hg(II) ions bind all three ApoE variants with approximately the same binding affinity, around 4–5 μM (Figure 2 and Table 1). This indicates similar binding sites with similar binding ligands in all protein variants. The residues that differ between the variants, that is, Cys112 residues in ApoE2 and ApoE3 and Cys158 in ApoE2, are therefore most likely not directly involved in Hg(II) coordination. Instead, the weaker binding to Hg(II) ions at acidic pH (Figure 2), where histidines are protonated, suggests that the His140 and His299 residues could be involved as Hg(II) binding ligands. Hg(II) binding was found to be non-cooperative, with Hill coefficients around 1, and multiple binding sites are likely present. Monovalent Hg(I) ions display much weaker binding affinities to all three ApoE variants, likely in the millimolar region (Figure 3), and induce virtually no change in the ApoE secondary structure (Figure 4). It therefore appears that the ApoE proteins do not have biologically relevant interactions with Hg(I) ions, which exist in reducing intracellular environments, but rather with Hg(II) ions, which exist in oxidizing extracellular environments. Bound Hg(II) ions induce minor but distinct structural alterations in all ApoE protein variants, interpreted as increased superhelicity and possibly located to the C-terminal domain, which contains lipoprotein binding and ApoE self-association sites and which has an intrinsic propensity to form coil-coiled interactions. The structural alterations are most pronounced in ApoE4 and least pronounced in ApoE2 (Figure 4). Thus, while the Hg(II) binding affinity is virtually the same for the three ApoE variants, the differences in structure induction between the three ApoE variants might conceivably be connected to the *APOE-*ε*4* gene being a risk factor in mercury intoxication but not the *APOE-*ε*2* and *APOE-*ε*3* genes. Future studies should clarify the binding sites for Hg(II) ions in the ApoE protein, the nature of the structural changes induced by the Hg(II) ions, and how these changes may affect the protein’s function(s).
